# ESR Bridges: CT builds bridges in coronary artery disease

**DOI:** 10.1007/s00330-023-10485-7

**Published:** 2024-01-31

**Authors:** Marc Dewey, José P. S. Henriques, Hristo Kirov, Rozemarijn Vliegenthart

**Affiliations:** 1 Charité—Universitätsmedizin Berlin, corporate member of Department of Radiology, Freie Universität Berlin and Humboldt-Universität zu Berlin, Berlin, Germany; 2 Berlin Institute of Health at Charité–Universitätsmedizin Berlin, Berlin, Germany; 3 Berlin University Alliance, Berlin, Germany; 4 DZHK (German Centre for Cardiovascular Research), partner site Berlin, Deutsches Herzzentrum der Charité (DHZC), Berlin, Germany; 5 Department of Cardiology, Amsterdam University Medical Centers, Amsterdam, The Netherlands; 6 Department of Cardiothoracic Surgery, Jena University Hospital, Friedrich Schiller University of Jena, Jena, Germany; 7 Department of Radiology, University Medical Center Groningen, University of Groningen, Groningen, The Netherlands

Imagine if we could non-invasively, reliably, and quantitatively detect coronary atherosclerosis and coronary luminal narrowing early in the development of coronary artery disease (CAD). We could fundamentally change the way CAD is diagnosed and treated. Such non-invasive and quantitative imaging of CAD using computed tomography (CT) is increasingly becoming a clinical reality worldwide, based on evidence generated in a multidisciplinary manner. The two key areas in which evidence has accumulated are the high diagnostic accuracy of CT when compared with invasive coronary angiography (ICA) as the reference [1–5] and improved patient outcomes using CT for comparative effectiveness in clinical trials [6–8].

This effort required technical developments towards multislice CT and cardiac-phase reconstruction [9, 10], as well as clinical evidence from multicentre registries [11–13], meta-analyses [1–5] and randomised controlled trials [6–8]. Three large multicentre clinical trials in stable chest pain patients with intermediate likelihood of CAD have been published (PROMISE, SCOT-HEART and DISCHARGE) and one large trial is ongoing (CLEAR-CAD). PROMISE indicated that CT is associated with a lower rate of death or non-fatal myocardial infarction compared to functional testing [6]. SCOT-HEART found fewer deaths from CAD or non-fatal myocardial infarction with CT and standard care compared to standard care alone at 5 years of follow-up [7]. DISCHARGE showed fewer major procedure-related complications in the CT compared to the ICA group [8].

## Three fundamental changes

Evidence has been generated primarily through close multidisciplinary collaboration between radiologists, cardiologists, cardiac surgeons, statisticians, epidemiologists, trialists and engineers. However, translating this evidence so that CT can truly begin to build bridges in CAD will require further multidisciplinary clinical collaboration to achieve real clinical impact through improved patient lives and well-being. What are the main arguments for integrating CT into the clinical pathways for patients with stable chest pain and suspected CAD? Based on the evidence described above, three fundamental changes in the approach to CAD are suggested [14] (Table 1).

**Table 1:** Three fundamental changes in the approach to CAD

Fundamental change	What CT provides	Evidence for change
1. **Better prevention of cardiovascular events** with the help of non-invasive imaging for the characterisation and quantification of coronary atherosclerosis	Early detection of coronary atherosclerosis by CT may lead to increased use of preventive therapy which results in fewer cardiovascular events	SCOT-HEART: more patients in the CT group used aspirin and statins at 5-year follow-up CAD-Man: higher statin adherence in the CT group at follow-up
2. **Individual determination of treatment strategy** becomes possible using non-invasive imaging for determining individually the presence and extend of CAD	Non-invasive assessment of obstructive CAD by CT for individualised decision-making about who may benefit most from revascularisation with additional functional tests	DISCHARGE: 50% greater use of additional invasive or non-invasive functional testing with 25% fewer revascularisation but similar angina reduction at follow-up
3. **Improved procedural planning** for the revascularisation strategy in patients with obstructive CAD	CT provides imaging biomarkers that could be used to guide revascularisation planning in individual patients in the future	Early, non-randomised studies; future randomised trials are needed

First, CT can better detect non-obstructive CAD and therefore early coronary atherosclerosis compared to ICA [8]. This may lead to increased use of preventive therapy with CT. In SCOT-HEART, more patients in the CT group used aspirin (52% vs 41%) and statins (59% vs 50%) at 5-year follow-up compared with the standard care group [15], which was associated with 50% reduction in CAD mortality or non-fatal myocardial infarction [7]. In addition, patients in the CT group of the CAD-Man trial had higher statin adherence (60%) compared with the ICA group (39%), resulting in greater cholesterol reduction at 3.3 years [16]. Better prevention contributes to a long-term reduction in myocardial infarction and stroke and is facilitated by the ability of CT to more accurately identify non-obstructive CAD when compared with ICA (36% versus 22%) [8].

Second, CT is well suited for determining the best treatment strategy in patients with suspected obstructive CAD [14]. The DISCHARGE trial showed a 50% greater use of additional invasive or non-invasive functional testing in the CT group compared with the ICA group, which was associated with a 25% reduction in revascularisation with no difference in angina reduction at 3.5 years of follow-up [8]. Non-invasive assessment of obstructive disease and individualised decision-making about who may benefit most from revascularisation is therefore an important part of the potential future success story of CT in CAD.

Third, improved procedural planning can be achieved in patients with obstructive CAD confirmed by CT. In patients with high-risk anatomy in whom revascularisation provides a prognostic benefit, the route of revascularisation (i.e. percutaneous or surgical or both) could in the future be determined based on quantitative coronary artery imaging biomarkers generated from CT [17–23]. It is just a matter of time before coronary artery bypass graft surgery can be planned based on CT, provided the image quality is high and taking into account additional functional testing and accepted severity (SYNTAX) criteria. For instance, an initial study showed that heart team decisions based on CT for the route of revascularisation were in high agreement when compared with ICA [24]. Furthermore, an ongoing nonrandomised study analyses the safety and feasibility of CT guidance for planning and executing coronary artery bypass grafting [25].

## Directions for future research

However, there are several limitations to the current evidence base for coronary CT. First, in patients with acute presentation, CT compared with established standard care reduced ICA rates but did not improve clinical outcomes [26]. Second, while artificial intelligence (AI) has potential to assess images and reduce workload of radiologists with high accuracy, there is limited implementation because little is known about which AI approaches might work best to individualise preventive (lipid lowering, anti-inflammatory and anti-thrombotic) therapy based on CT findings [17, 18]. Third, the current evidence in favour of CT is limited to patients with stable chest pain and an intermediate likelihood of obstructive CAD [14], suggesting that further multidisciplinary research is needed to assess patients on other ends of the clinical spectrum in which the use of CT in suspected CAD can be expected to be beneficial.

Despite these limitations, it is clear that multidisciplinary research has already built important bridges in the diagnosis and treatment of CAD and can serve as a bridgehead to further link radiology with the other disciplines to undertake even more ambitious efforts of non-invasive and quantitative cardiovascular imaging research to improve the care of our cardiovascular patients in the future.

## Recommendations for clinical practice and directions for future research

- *Better prevention of cardiovascular events* with the help of non-invasive imaging for the characterisation and quantification of coronary atherosclerosis. Early detection of coronary atherosclerosis by CT may lead to increased use of preventive therapy which results in fewer cardiovascular events.
- *Individual determination of treatment strategy* becomes possible using non-invasive imaging for determining individually the presence and extent of CAD. Non-invasive assessment of obstructive CAD by CT for individualised decision-making about who may benefit most from revascularisation with additional functional tests.
- *Improved procedural planning* for the revascularisation strategy in patients with obstructive CAD. CT provides imaging biomarkers that could be used to guide revascularisation planning in individual patients in the future.

## Declarations

### Guarantor

The scientific guarantor of this publication is Marc Dewey.

### Statistics and biometry

No complex statistical methods were necessary for this paper.

### Methodology

• commentary
